# Supplementary material for: Increasing extreme precipitation variability plays a key role in future record-shattering event probability
Source: Commun Earth Environ. 2024 Sep 3;5(1):482. doi: 10.1038/s43247-024-01622-1 (PMC11371648; doi:10.1038/s43247-024-01622-1)
Supplement: Supplementary file 1 — Supplementary information [file 43247_2024_1622_MOESM1_ESM.pdf]

Supplementary information to
"Increasing extreme precipitation variability plays key role in future record-shattering event probability"

Iris de Vries[1,*], Sebastian Sippel[2], Joel Zeder[1], Erich Fischer[1] & Reto Knutti[1]

[1]Institute for Atmospheric and Climate Science, ETH Zürich, Switzerland, *iris.devries@env.ethz.ch
[2]Leipzig Institute for Meteorology, Leipzig University, Germany

S1 Supplementary section 1: Event comparison: observational bias and additional simulated event

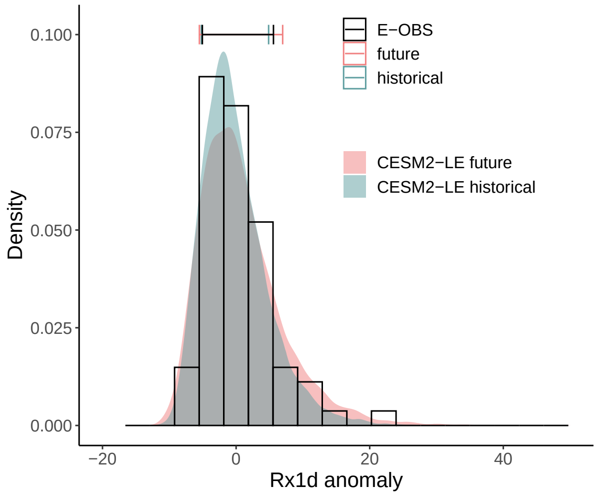

SI Figure S1: Distribution of regionally averaged BNLG Rx1d anomalies in CESM2-LE (shading) and E-OBS (black histogram). Top bars indicate one standard deviation around the mean. Anomalies are computed relative to 1950–2020 averages and, for CESM2-LE, within each member individually.

Fig. S1 shows that the observational Rx1d anomalies lie within the range of the simulated anomalies in CESM2-LE. Also the spread of observational anomalies is very similar compared to simulated anomalies, as indicated by the bars at the top which show the standard deviation around the mean. The shift to higher, more variable values in future years in CESM2-LE is visible.

Fig. S2a-d show the meteorological situation associated with the most extreme event simulated by CESM2-LE in the historical period (1850-2022). The circumstances of this simulated record-shattering event are, once again, similar to those of the 2021 European Floods, shown in main Fig. 1b-e. Fig. S2e shows all simulated MJJAS Rx1d values and its corresponding mean sea level pressure anomaly on the same day, averaged over the BNLG region (red box in Fig. S2a-d). This figure confirms that the vast majority of simulated MJJAS Rx1d events is associated with negative surface pressure anomalies (surface lows), as we also see for the simulated and observed record-shattering events. The coloured dots show the record-shattering events from 1950 onwards (since record-shattering events early in the timeseries are more likely to be part of the 'spin up' phase of setting the baseline), which exhibit a clear negative correlation between event intensity and depth of the surface low (significantly correlated with a Pearson correlation coefficient of -0.22). This corroborates that the meteorological conditions shown in main Fig. 1 are representative of extreme precipitation events in the BNLG region in extended summer. The colour of the dots also hints at an intensification of record-shattering events as the end of the century is approached.

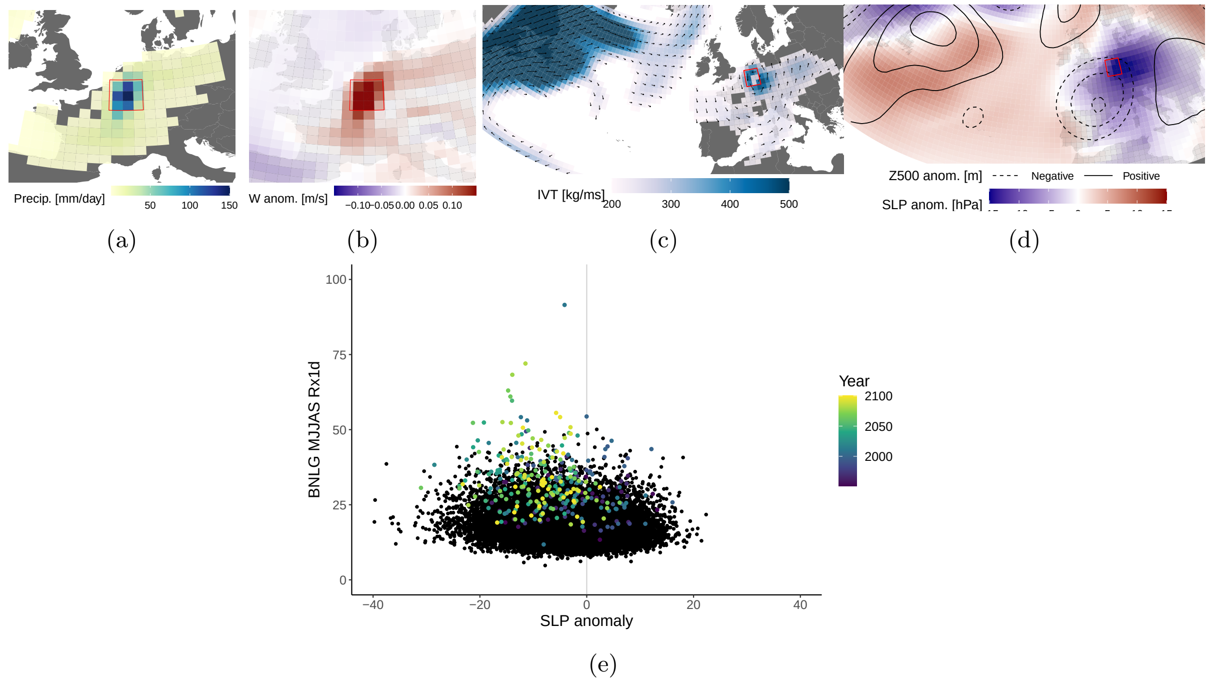

SI Figure S2: (a-d): as main Fig. 1f-i but for the most extreme historical (1850-2022) record-shattering event simulated by CESM2-LE (July 17th 2010). (e) shows climatology of Rx1d and sea level pressure (SLP) for local summer (MJJAS) Rx1d events in the Benelux/South Germany (BNLG) region (red box in a-d). Precipitation and SLP values are first spatially averaged for the region, then the maximum is extracted to obtain Rx1d. SLP anomalies are determined within each individual member by subtracting the multiyear (1850-2100) daily mean SLP from each respective day.

S2 Supplementary section 2: Validation GEV fits

The validity of the results presented in the main text depends to a large extent on the validity of the GEVs fitted to the data. It is important to keep in mind that the quantitative absolute probabilities shown in main Sect. 2.5 are strongly dependent on the length of the timeseries and the emission scenario input into the climate model, and therefore these values are to be interpreted in the context in which they were generated. Yet, the probability ratios and qualitative record-shattering behaviour are instructive to explore the relative response of record-shattering precipitation given the simulation conditions. To confirm that the results are reliable, the methods, which heavily rely on fitted GEV distributions, are validated below.

Supplementary Fig. S3-S5 show GEV metrics for 20 arbitrarily chosen grid cells, indicated on the map in Supplementary Fig. S3. First, QQ-plots are shown which serve as a visual test of goodness of fit: the closer the points are to the 1-1 line, the better the fit. We see that the fit is good for the grid cells shown, but towards the very high percentiles (top right corners), deviations from the 1-1 line. This is expected given the difficulty associated with accurately fitting the tail of heavy tailed distributions [1, 2].

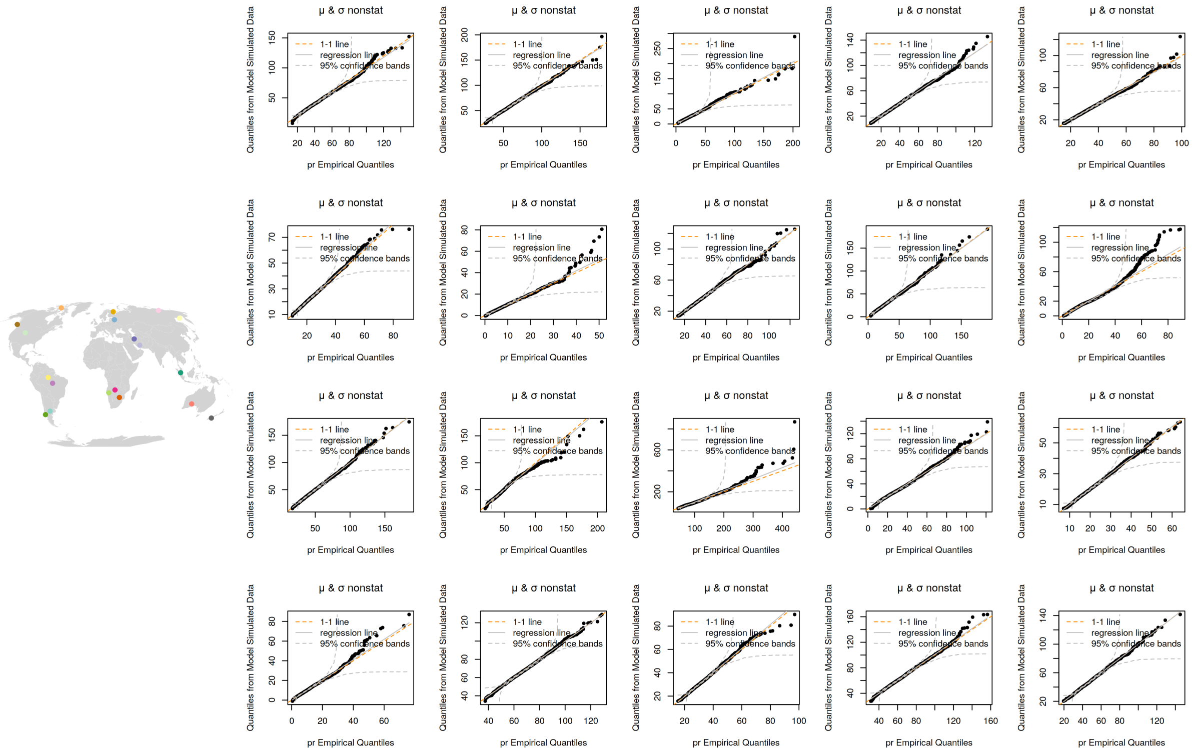

SI Figure S3: QQ-plots of GEVs fit to MJJAS 5yRx1d of 20 arbitrarily chosen grid cells, shown to the left

Supplementary Fig. S4 and S5 show tests of different GEV setups, in order to validate the GEV setup choice we employ in this study. When fitting GEVs to data, the (non-)stationarity and the covariates of the three GEV parameters are chosen by the user. In main Sect. 4.3 we describe the GEV setup we use, with non-stationary location and scale parameter μ and σ. There are, however, other GEV setups possible. We assess four different GEV setups:

1. **All stat**: all three GEV parameters are prescribed to be stationary, meaning that for each grid cell, one GEV is fit to the data from 1850-2100.

2. **μ/loc nonstat: only the location parameter μ is non-stationary and varies linearly with its covariate: the smoothed ensemble mean of 5yRx1d. This setup effectively assumes that the variability does not change.**

3. **μ/loc + σ/sigma nonstat: both the location parameter μ and the scale parameter σ are non-stationary and vary linearly with the smoothed ensemble mean (μ) and standard deviation (σ) of 5yRx1d. This is the setup we use in the study.**

4. **All nonstat: location parameter μ, the scale parameter σ and shape parameter ξ are non-stationary and vary linearly with the smoothed ensemble mean (μ) and standard deviation (σ and ξ) of 5yRx1d. This setup assumes every aspect of the distribution varies with time.**

Supplementary Fig. S4 shows Akaike and Bayesian information criteria (AIC/BIC) for the different GEV setups. Based on the notion that the statistical models we propose are imperfect representations of the true data generating process, the AIC and BIC of a certain model represent the amount of information lost relative to the true distribution if that model is used to estimate the distribution. Hence, the lower the AIC/BIC value, the more accurate the model. We see that there is only a small difference in AIC/BICs for different model setups, but the general tendency for all 20 arbitrary grid cells is lowering of the AIC/BIC as non-stationarity is added to the model, however, between setup 3 and 4 (see above) there is little improvement.

This is further confirmed by Supplementary Fig. S5, where we show the p-values of likelihood ratio tests comparing the different models above in order of increasing complexity. The x-axis shows the hypothesis of the test, always being "less complex model is better than more complex model". The p-value indicates

the probability of observing the GEV-fitting results under this hypothesis. Thus, if the p-value is very small, say below 0.05, there is a very small probability that the hypothesis on the x-axis is true. In other words, if the p-value is very small, we expect the more complex GEV setup to be significantly better. We observe that the p-values are, minus one exception, all below 0.05 for the first two hypotheses, meaning adding non-stationarity in the location and scale parameters significantly improves the GEV fits. For the last hypothesis, which tests the improvement of adding non-stationarity in the shape parameter, we see a majority of the grid cells with one of both seasons featuring p-values larger than 0.05. This indicates that the added value of non-stationarity in the shape parameter is not significant in most cases. In combination with the difficulty of estimating the shape parameter, it is therefore justified to keep it constant.

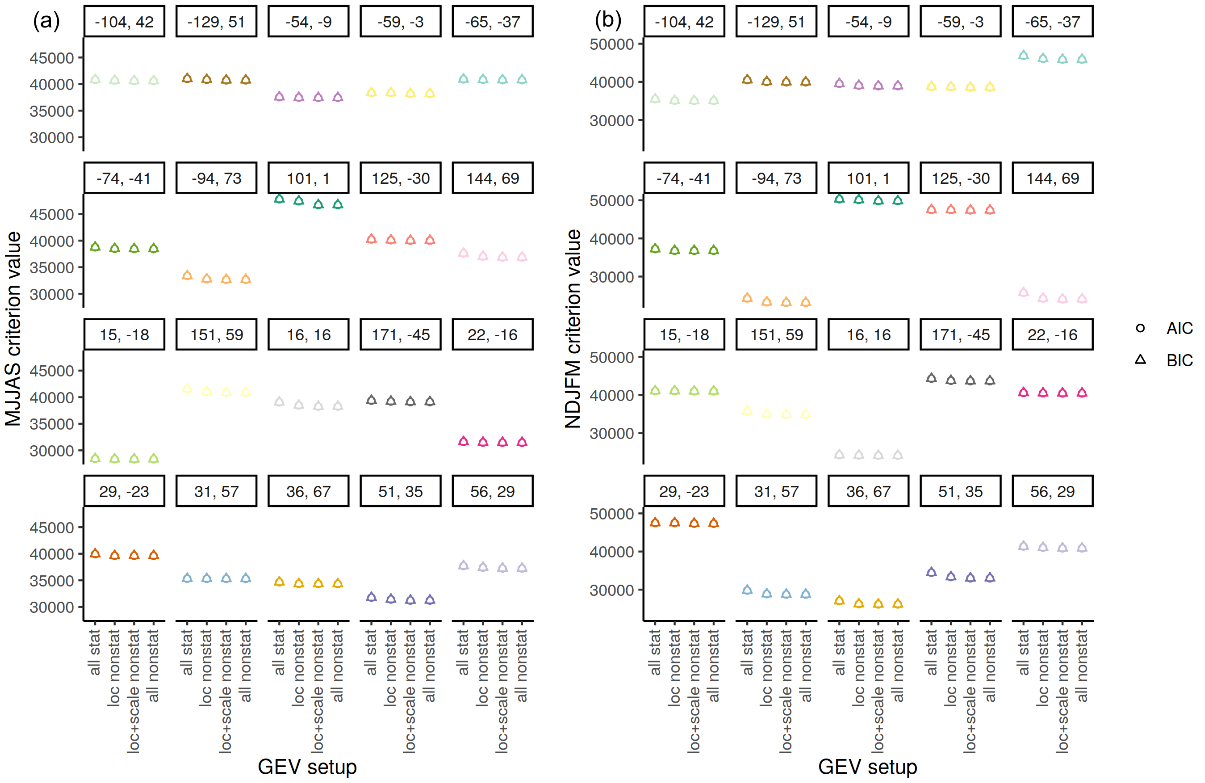

SI Figure S4: Information criteria (IC) for the different GEV setups (x-axis) for the same arbitrary 20 gridcells as shown above, (a) MJJAS and (b) NDJFM. The lower the value of the IC, the better the fit of the corresponding distribution.

Supplementary Fig. S6-S8 show QQ-plots as in Supplementary Fig. S3 for MJJAS 5yRx1d in the three regions we discuss in the main text, where the subplots all represent one grid cell. Also here we see satisfactory goodness of fit of the GEVs, with however increasing uncertainty/inaccuracy in the tails. The high quantile deviation from the 1-1 line is largest in Lagos and Pakistan. This could imply that the tropical/monsoonal character of the most extreme precipitation events in those regions leads to events that effectively follow another distribution than the bulk. In statistical terms, 5-year block maxima in these regions are not extreme enough to be max-stable. Increasing block size further, however, would reduce the sample size too much.
The fact that the GEV-fits are not perfect does not interfere with the purpose of this study, which is to show how changes to the precipitation distribution impact record-shattering precipitation probabilities, rather than to predict the true record-shattering probability. The GEV-fits are good enough to represent extreme precipitation behaviour, judging from the agreement of the results based on generated data and CESM2 data. Hence, we are confident that the lower goodness of fit of the GEV in the highest quantiles does not affect the main findings of this study.
Lastly, Supplementary Fig. S9 shows the information criteria (a-c) and likelihood ratio tests (d-f) for the three regions, see above for an explanation of their interpretation. The spread across gridcells in one region is represented by the boxplots. Also for the three regions, we see that the information criterion values decrease with increasing GEV complexity, however, little information is added by adding non-

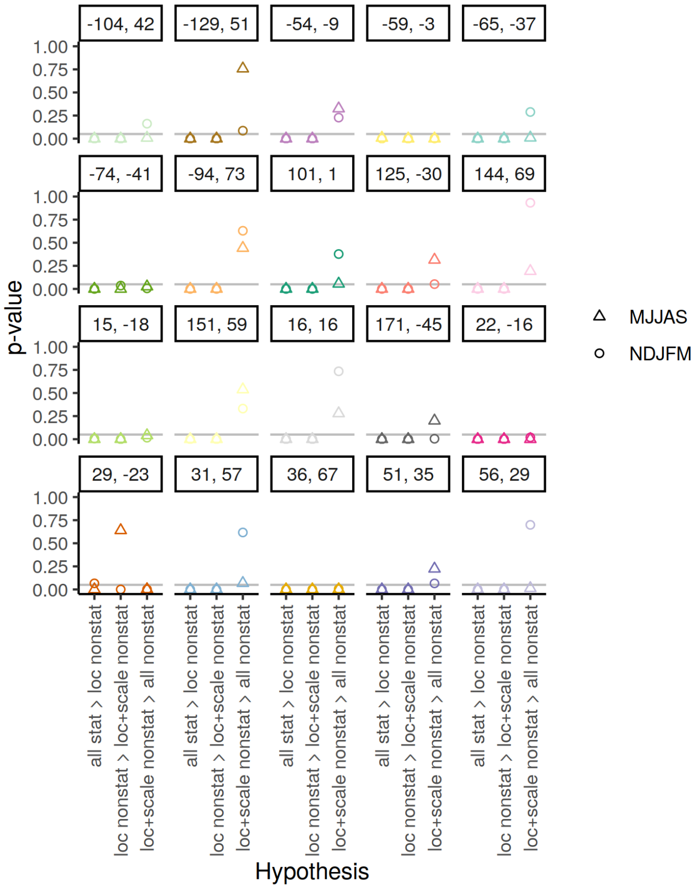

SI Figure S5: Likelihood ratio test for the different GEV setups for the same arbitrary 20 gridcells as shown above. If the p-value is smaller than a threshold, here chosen to be 0.05, depicted by the grey horizontal line, the hypothesis on the x-axis can be rejected at a $1-$p-value confidence level. If the p-value lies above the grey line, there is no significant difference in suitability of the compared GEV-setups.

stationarity in the shape parameter. Also the likelihood ratio tests show that non-stationarity in location and scale parameters improves the GEV significantly, whereas non-stationarity in the shape parameter does not do so across regions and seasons.

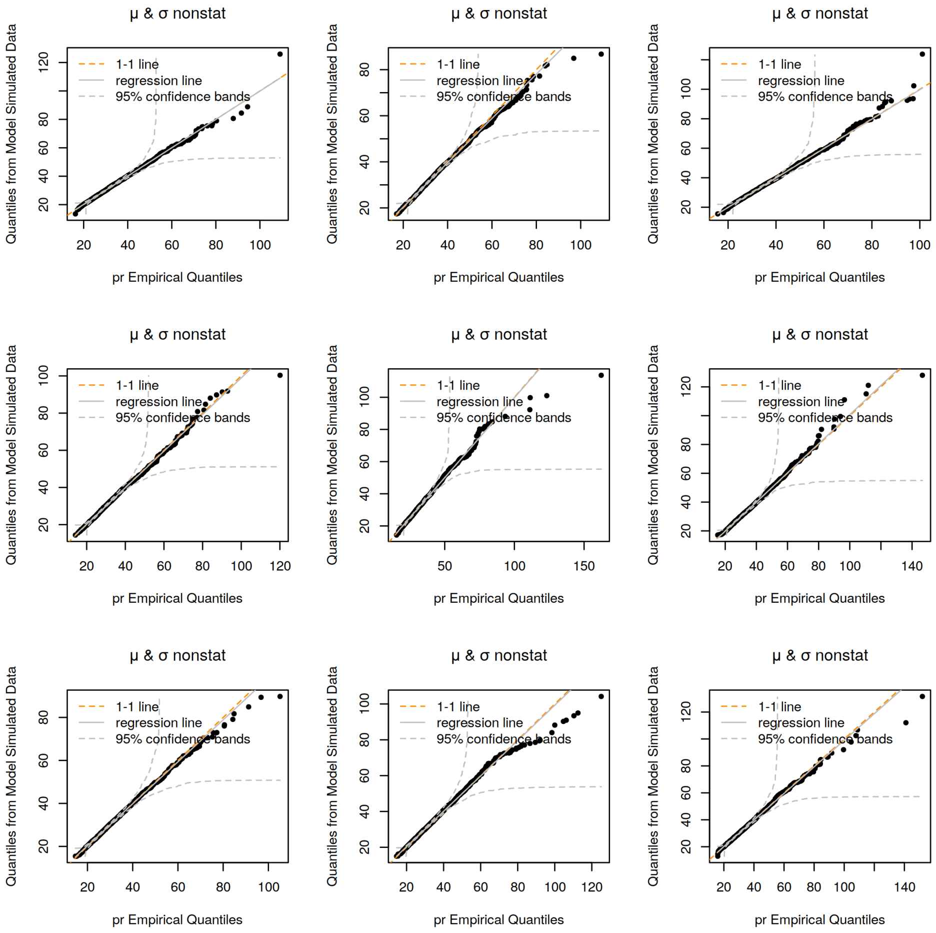

SI Figure S6: BNLG MJJAS 5yRx1d QQ-plots of GEV-fits

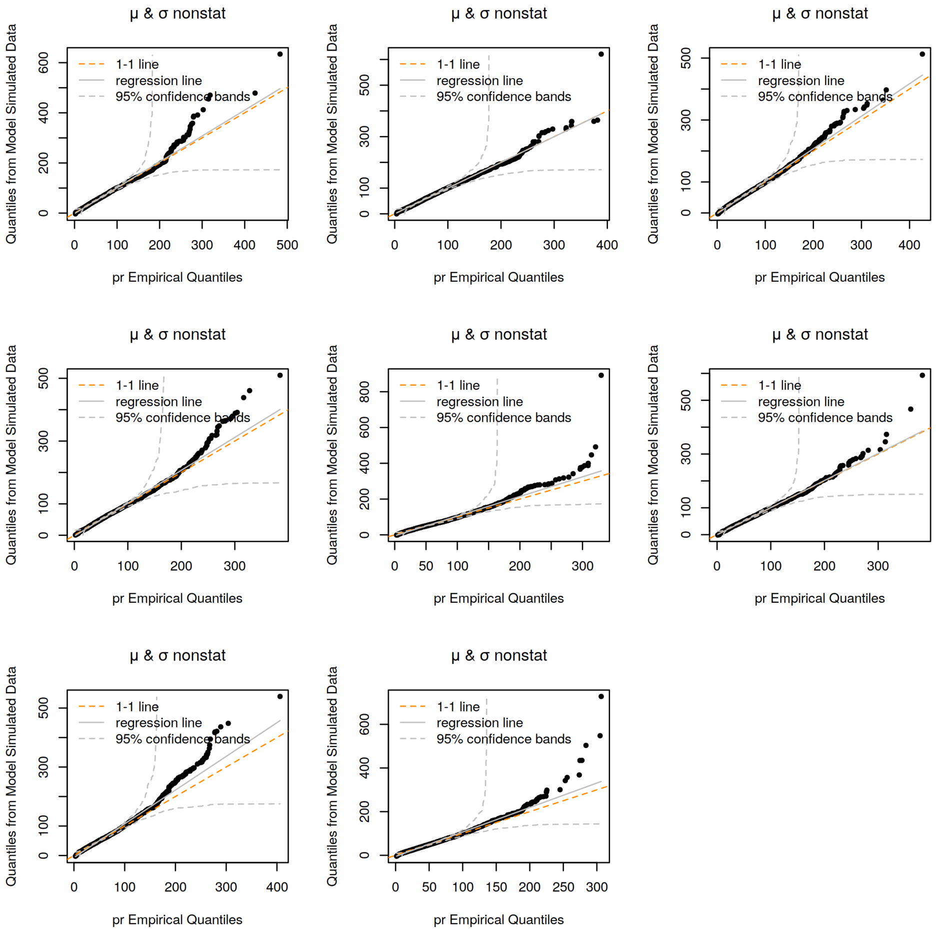

SI Figure S7: Pakistan MJJAS 5yRx1d QQ-plots of GEV-fits

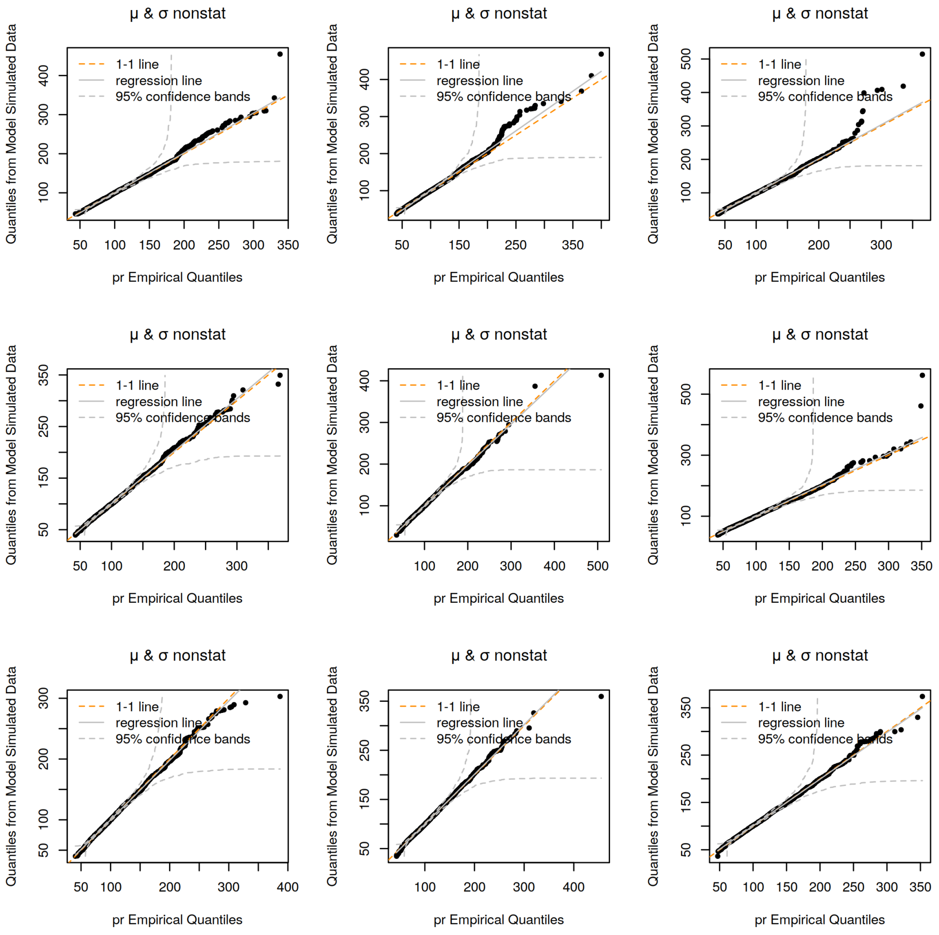

SI Figure S8: Lagos MJJAS 5yRx1d QQ-plots of GEV-fits

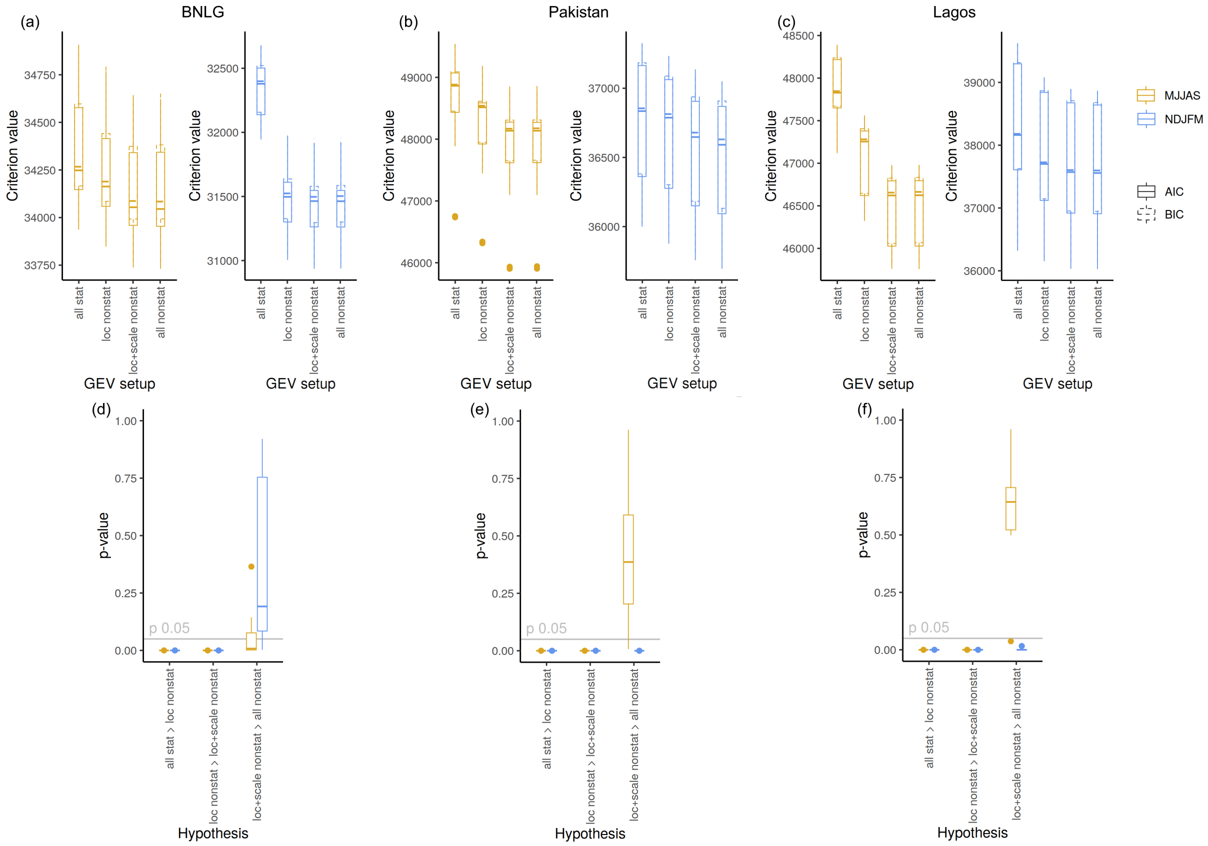

SI Figure S9: Information criteria (a-c) as in S4 and likelihood ratio tests (d-f) as in S5 for the indicated different setups of GEVs fitted to the regions of interest. Boxplots show the spread of the eight or nine grid cells that make up each region (see main Sect. 4.6 for region definitions).

S3 Supplementary section 3: Additional material on global record-shattering probabilities in CESM2-LE

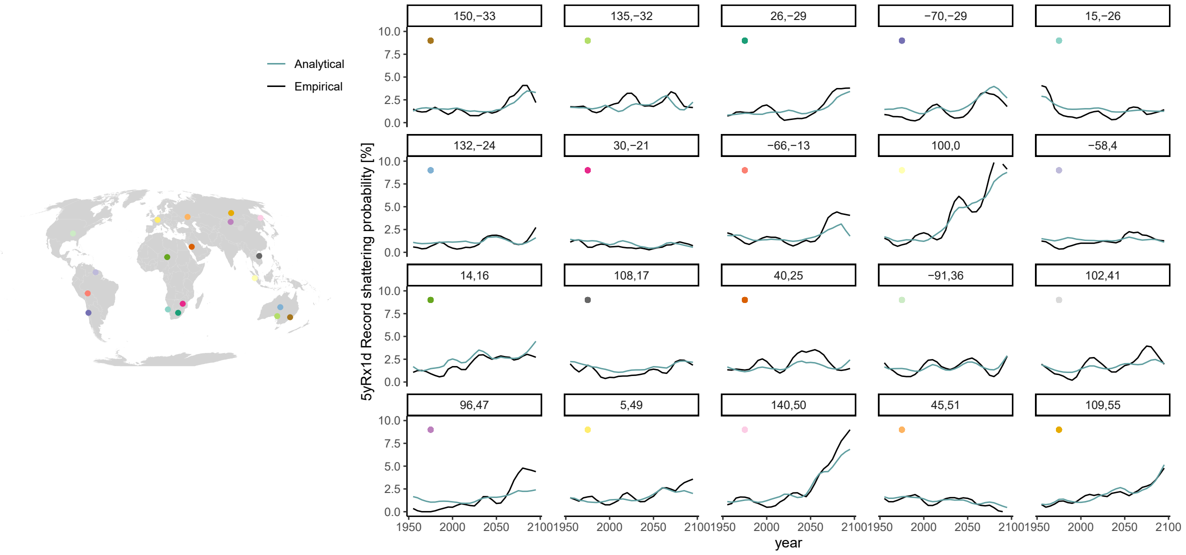

SI Figure S10: Timeseries of empirical and analytical 5yRx1d record-shattering probabilities (P_{CCe} and P_{CCa}) at randomly chosen indicated single grid cells. Titles of subplots indicate longitude and latitude of the corresponding grid cells

Fig. S10 shows that timeseries of empirical and analytical 5yRx1d record-shattering probabilities, determined as described in main Sect. 2.2, 4.4, and 4.5, are highly congruent for the 20 randomly selected grid cells shown. For other sets of randomly selected grid cells agreement is similar.
Fig. S11 shows the climatological 5yRx1d, averaged for the 1850-1949 period, before the emergence of (strong) climate change effects in panels a-d. These maps show that the probability ratios shown in main Fig. 2 are primarily high where 5yRx1d has high climatological levels and variability. In addition, the linear change summed over the present and future period (1951-2100) in both mean 5yRx1d and 5yRx1d variability (standard deviation) are clearly strong predictors for high probability ratios. The mean trends (e-f) are normalised with respect to the local historical ensemble SD, and thus represent the trend relative to the natural variability. For most regions, the highest trends occur where climatological levels are high as well, for both mean and variability (pattern intensification). However, in e.g. the northern hemisphere in local winter (NDJFM), the normalised mean trend is large (f), despite low climatological levels (b). Such regions with large normalised mean trends, clearly appear in the probability ratios maps in main Fig. 2, indicating that the trend relative to the variability is a metric of interest for record-shattering probabilities, as corroborated by main Fig. 3.
Fig. S12 shows the 2070-2099 average of the absolute 5yRx1d record-shattering probabilities in CESM2-LE. For P_{CC}, i.e. record-shattering probabilities in a climate with climate change according to SSP3-7.0, the empirical (a, c) and analytical (b, d) probabilities are shown for both extended seasons (MJJAS and NDJFM). P_{ref}, i.e. record-shattering probabilities in a stationary, pre-industrial climate (1850-1949 mean climate) is always analytical (e, f). The absolute probabilities exhibit the same patterns as the probability ratios shown in main Fig. 2 and reach maximum values of over 10% locally. A 10% record-shattering probability in a grid cell means that for that single grid cell, the probability of shattering the historical 5yRx1d record in any given 5 year window in 2070-2099 is 1 in 10. P_{ref} is uniformly very small, varying between 0.5 and 1% for the largest part of the global land (note the colour scale). This is in line with theory: $1/t$ for 5yRx1d record breaking in 2070-2099 $\approx 2\%$, and for record shattering this probability is even lower.
Visually we can distinguish a relatively clear inverse correlation between P_{CC} and P_{ref}: where P_{CC} is high, P_{ref} is generally low. This characteristic contributes to larger probability ratios. From a statistical point of view, keeping Eq. (2) in the main text in mind, this makes sense. For P_{ref} both the probability density function $f(x)$ and the cumulative distribution function $F(x)$ are constant with time. P_{ref} is thus high if either the probability of hitting $x + c$ is high ($f(x + c)$), or the probability that x has not been exceeded up to the present is high ($\prod F(x)$). The former will generally be true for wide distributions

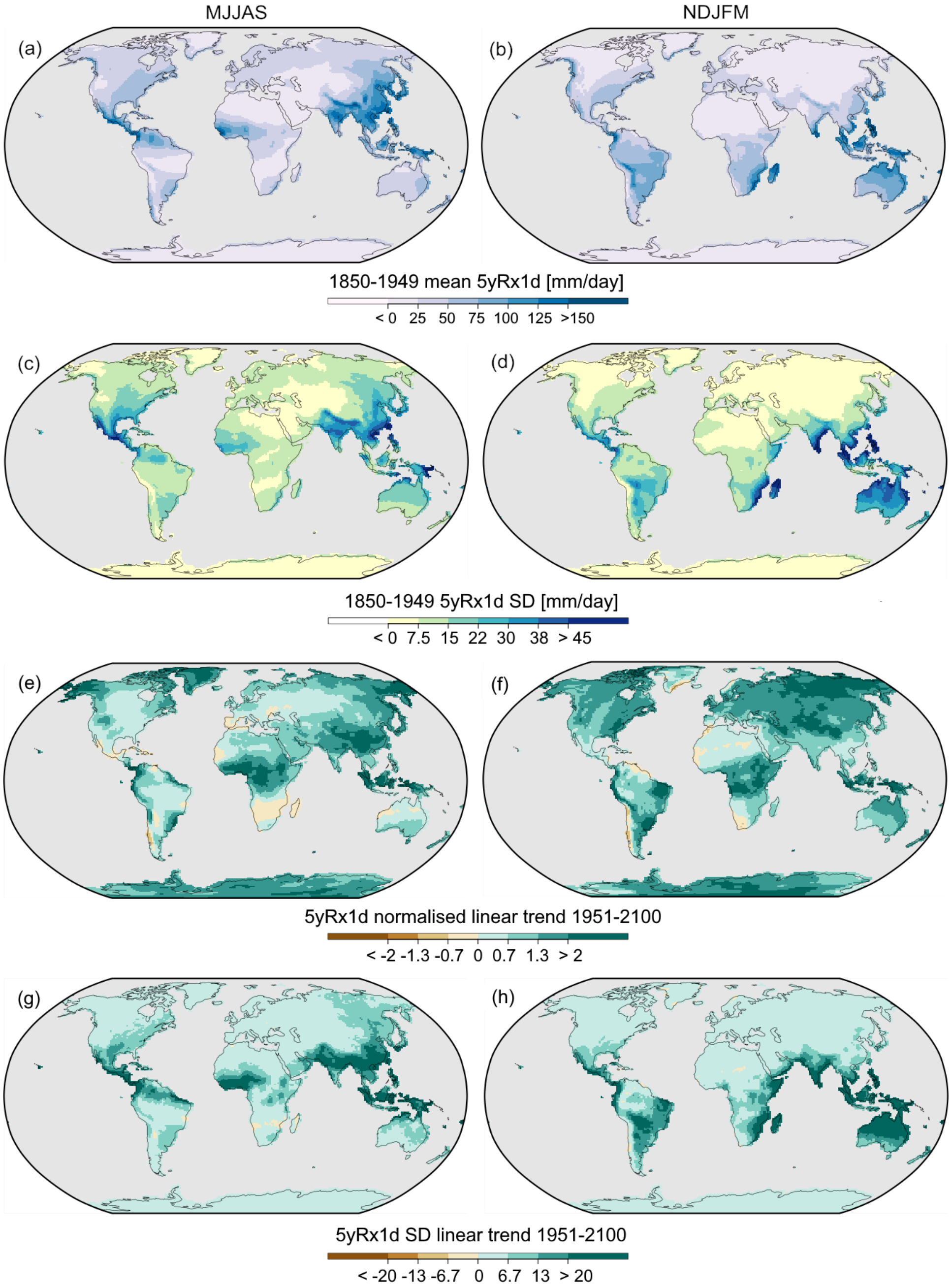

SI Figure S11: 5yRx1d 1850-1949 mean ensemble mean for May-September (MJJAS) and November-March (NDJFM) (a, b); 5yRx1d 1850-1949 mean ensemble standard deviation (SD)(c, d); 5yRx1d 1951-2100 ensemble mean linear change over full period (150 years) normalised w.r.t local 1850-1949 mean ensemble SD (e, f); 5yRx1d 1951-2100 ensemble SD linear change over full period (150 years) (g, h).

with long right tails, and the latter will be true for narrow distributions with short right tails. The value of P_{ref} will be a trade-off of these two opposing tendencies, and depends on their relative dominance. It makes intuitive sense that the tropical and monsoon regions where precipitation is systematically high, exhibit a small probability that x has not been exceeded up to the present ($\prod F(x)$), thus showing small P_{ref}. In northern hemispheric midlatitudes in MJJAS (summer), on the contrary, precipitation is systematically not that high, keeping $\prod F(x)$ at reasonably high levels. Yet, the sporadic occurrence of heavy convective summer storms results in a thin, long, right tail leading to relatively high probabilities of exceeding $x + c$ (high $f(x + c)$) and thus high P_{ref}. Northern hemispheric winter (NDJFM), featuring precipitation primarily from large scale weather systems, has a less chaotic nature, small $f(x + c)$ and thus small P_{ref}. Lastly, the dry subtropics naturally have very high $\prod F(x)$ values due to lack of rain,

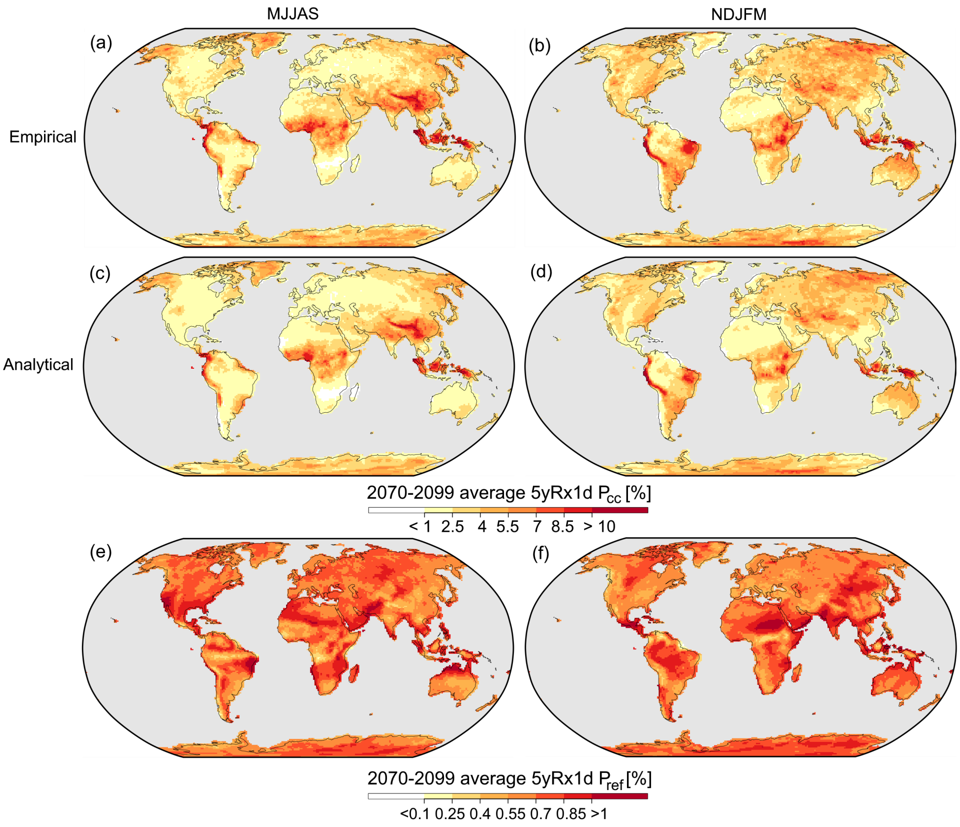

SI Figure S12: Absolute seasonal 5yRx1d record-shattering probabilities averaged over 2070-2099, as projected by CESM2-LE for the SSP3-7.0 scenario P_{CC}, empirical (a, b) and analytical (c, d), and in a reference preindustrial climate P_{ref} (e, f). See main Sect. 2.2, 4.4, and 4.5 for details on the computation of the variables shown.

leading to high P_{ref}.
Interestingly, the effects of climate change on the probabilities also depends on the relative changes in $f(x+c)$ and $\prod F(x)$. Increasing mean and variability increase $f(x+c)$ instantaneously, but, over time, decrease $\prod F(x)$. The anticorrelation between P_{ref} and P_{CC} and the findings in the main text suggests that climate change is effective at increasing record-shattering probabilities in a background climate with low P_{CC} due to low $\prod F(x)$ by shifting up the distribution $f(x+c)$ through changes in the mean or changes in variability.
The maps of probability ratios resulting from trends in either location parameter μ or scale parameter σ only, shown in Fig. S13, indicate the importance of both distributional changes, yet highlight that the scale parameter plays an especially large role. Regions where probability ratios are large exhibit large contributions from trends in both parameters, however, the σ-trend contributions cover a larger area fraction and are generally larger. A few distinct locations show higher μ-trend contributions, mostly associated with mountain ranges (Andes, Himalayas, Papua New Guinea). The strong contribution of both trends in these mountainous regions might be explained by positive effects of warming on both water vapour content and orographic lift. Also the central and northwestern parts of the Eurasian continent stand out for the strong μ-trend contributions in local winter. The nature of precipitation (large scale systems) and polar amplification might play a role here.

S4 Supplementary section 4: Robustness of probability ratios across CMIP6 models

Fig. S14 shows the probability ratios for the four individual CMIP6 models, for comparison with CESM2-LE in main Fig. 2. Fig. S15a and b show the analytical seasonal probability ratio pattern averaged over CESM2-LE, ACCESS-ESM1-5, MPI-ESM1-2-HR, MPI-ESM1-2-LR, and UKESM1-0-LL, and c and d show the relative root mean squared error (RMSE) in probability ratios as a measure of the

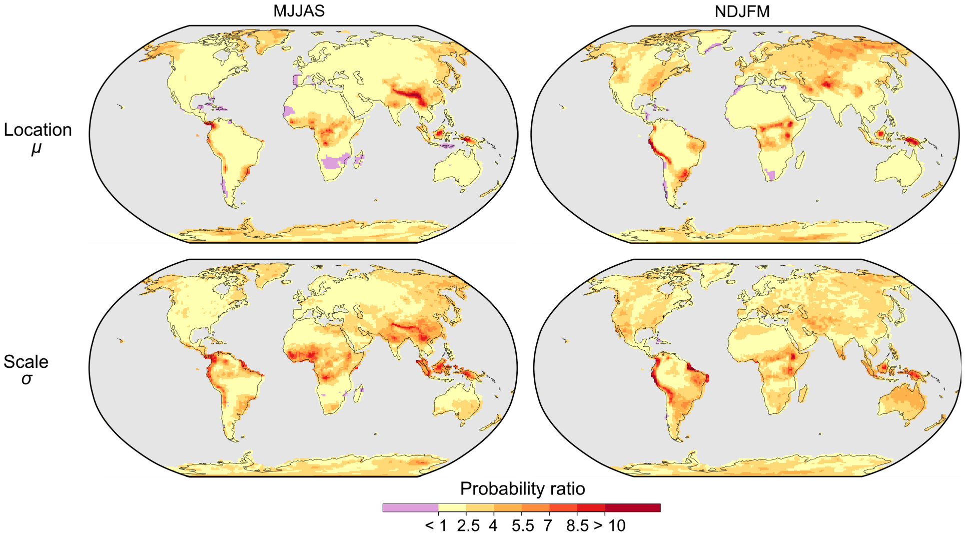

SI Figure S13: Breakdown of 2070-2099 average 5yRx1d record-shattering probability ratio into components due to trends in location parameter μ and scale parameter σ of the GEV distribution. Refer to main Fig. 2 for the full probability ratios and to main Sect. 2.2, 2.3, 4.4, and 4.5 for details on the computation of the variables shown.

difference between the individual models' probability ratios and the multi-model mean. The smaller the latter (darker green), the higher the agreement of the models on the mean value. MPI-ESM1-2-HR and MPI-ESM1-2-LR dominate the average around 15° North (the transition region between seasonally wet savanna to the south and dry Saharan climate to the north) with very high values, likely due to very dry climatology resulting in non-physically high probability ratios (dividing by very small numbers). Therefore, Fig. S4e-h show the multi-model mean obtained when the MPI-models are excluded. The multi-model mean pattern is highly similar to the CESM2-LE single-model pattern, indicating the regional variations in precipitation record-shattering probability ratios are robust. The mean absolute deviation is largest where probability ratios are largest, however, when the MPI models are not included, the median RMSE for the global land decreases.
Lastly, Fig. S16 shows the component breakdown for all the CMIP6 models, providing the full global fields that underlie the zonal means shown in main Fig. 2.

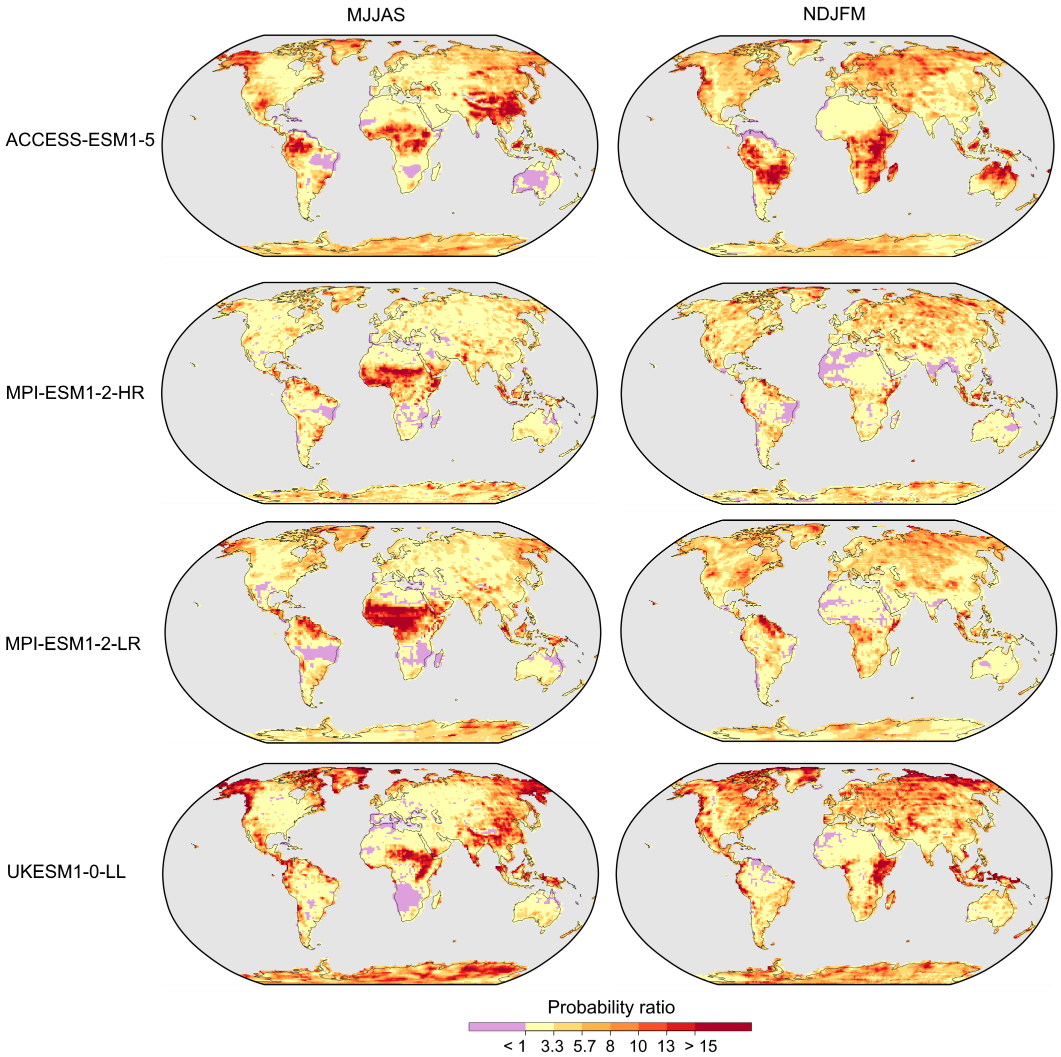

SI Figure S14: Probability ratios as in main Fig. 2 for each of the four CMIP6 models listed in main Sect. 4.1, for MJJAS and NDJFM seasons.

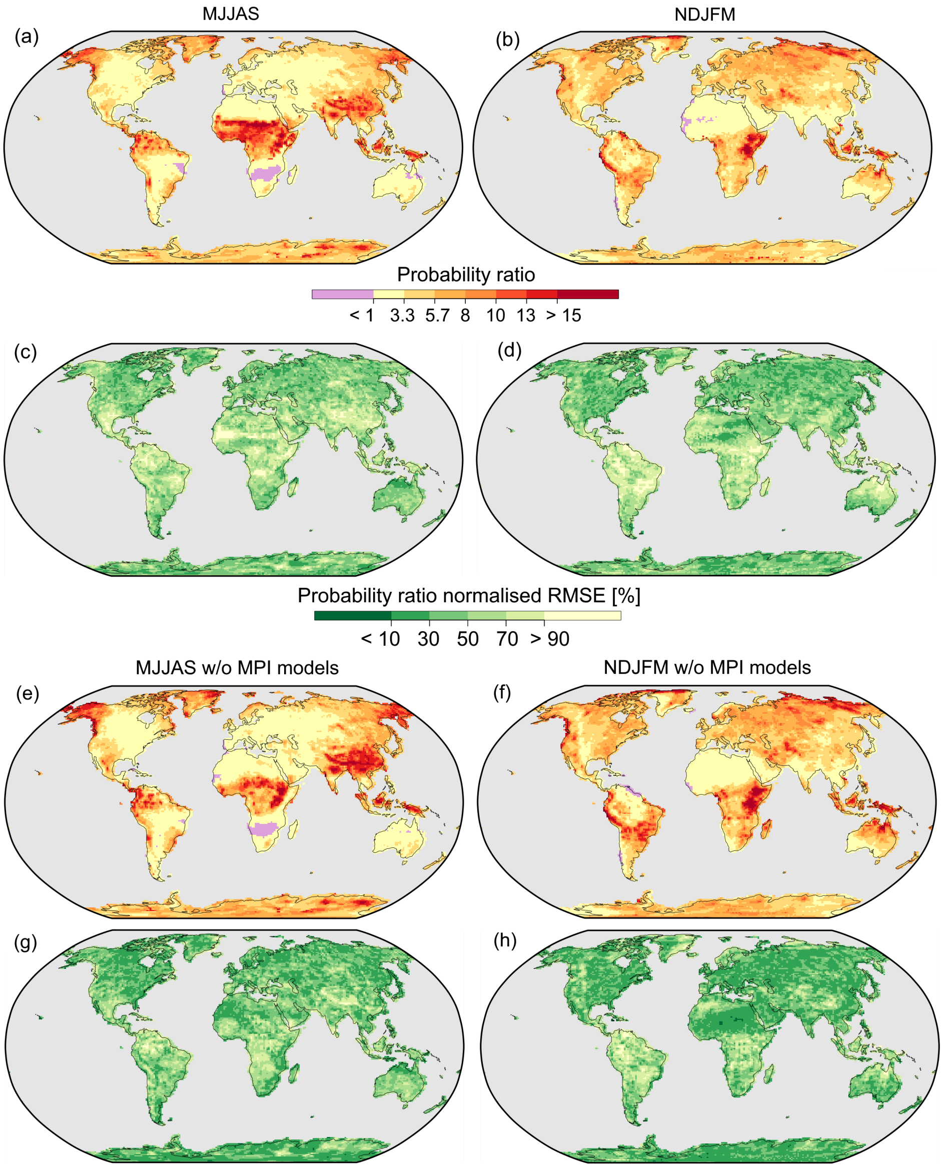

SI Figure S15: Probability ratios as in main Fig. 2 averaged over CESM2-LE and the four CMIP6 models listed in main Sect. 4.1, for MJJAS and NDJFM seasons (a, b). Probability ratio mean absolute deviation (mean of model deviations from the multi-model mean) across the five-model ensemble (c, d). Mean probability ratios and absolute deviation for MJJAS across all models except MPI-ESM1-2-HR and MPI-ESM1-2-LR (e, f).

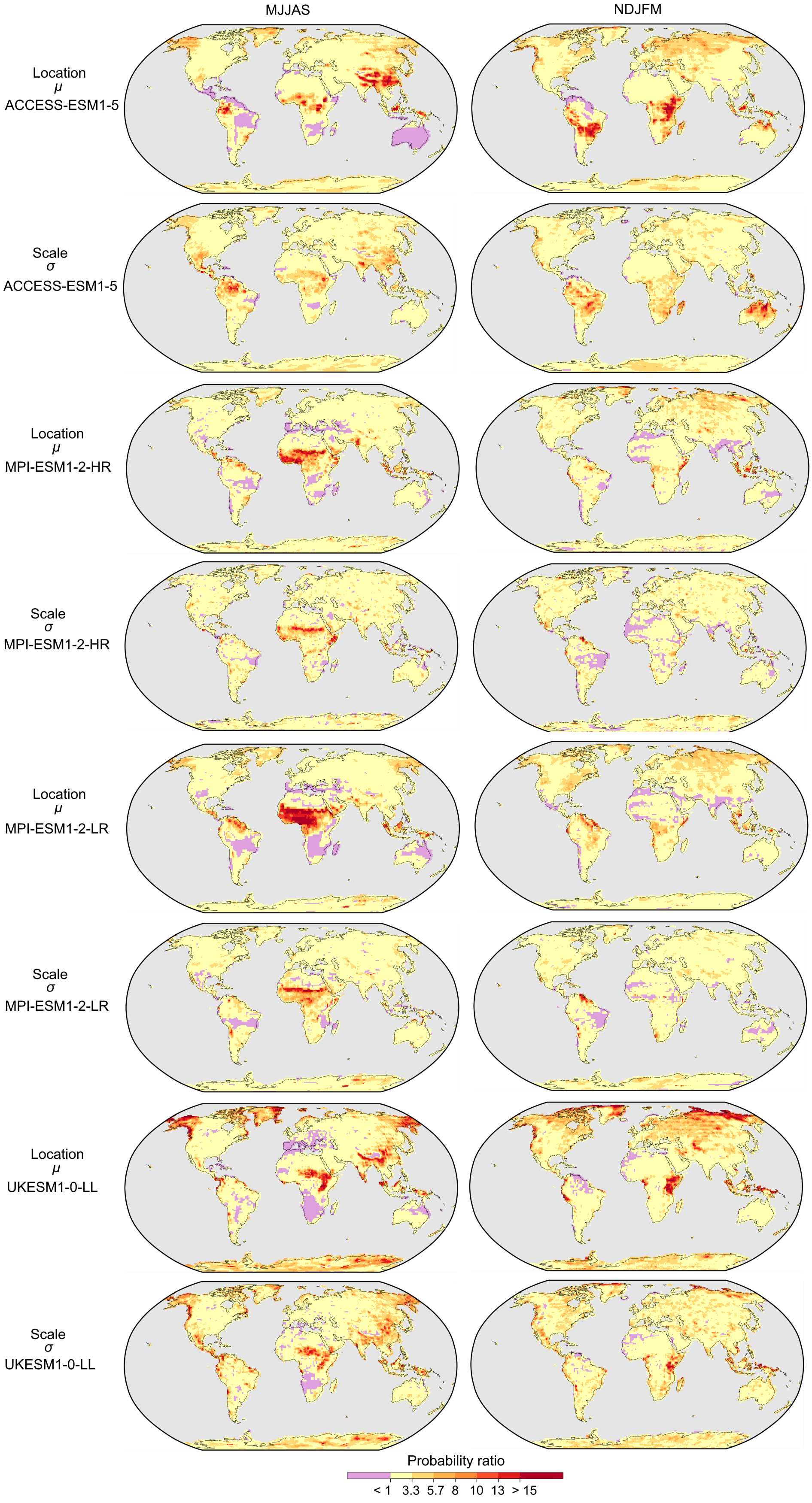

SI Figure S16: Breakdown of probability ratios into components due to changes in location and scale parameter, as in Supplementary Fig. S13, for each of the four CMIP6 models listed in main Sect. 4.1, for MJJAS and NDJFM seasons.

S5 Supplementary section 5: Sensitivity GEV-based analytical solution to ensemble size

Fig. S17 shows the sensitivity of the GEV parameter estimates to the ensemble size of the CESM2-LE ensemble. This sensitivity is assessed to support the claim that record-shattering probability ratios can be estimated in smaller ensembles by the use of non-stationary GEV distributions, in main Sect. 2.3. That statement only holds if GEV parameter estimates are sufficiently robust across ensemble sizes.
We assess this robustness by fitting non-stationary GEV distributions to the global data, as described in main Sect. 4.3, using the full CESM2-LE ensemble of 100 members, as well as random selections of 50, 30, 20, 10, and 5 ensemble members. This results in GEV parameter estimates for all these ensemble sizes for all land grid cells. To visualise this in a somewhat concise manner, we show boxplots of the GEV parameter estimates and their 95% confidence intervals (CIs) for all ensemble sizes for MJJAS 5yRx1d. We show two $\approx 10°$ latitude bands, one in the Northern Hemisphere midlatitudes (black outline) and one in the tropics (light grey outline), representing two different extreme precipitation regimes. Two 5-year windows are shown; the 1950-1954 window to reflect the distribution at a time with little apparent climate change effects, and the 2094-2099 window where climate change effects are largest (note, since ξ is not allowed to vary with time, we only show one window for ξ). The left panel of this figure shows parameter estimates. For μ and σ the boxplots are visually almost identical for all ensemble sizes and both latitude bands. ξ is most sensitive to ensemble size, and shows a small decreasing trend as ensemble size decreases beyond 20 (trend across differently coloured boxplots), and an increase in spread across the latitude band (height of bar). The ensemble size sensitivity does not differ qualitatively between the two zonal locations, yet, the higher precipitation at tropical latitudes also seems to lead to a stronger increase in uncertainties in μ and σ with decreasing ensemble size. Between the two 5-year windows we see the expected effects of climate change reflected in increasing μ and σ, especially in the tropics. Sensitivity to ensemble size does not change with climate change.
The right panel shows the magnitude of the 95% CIs around the mean of the GEV parameter estimates in the same way (generated with the extRemes package, functions *fevd()*, *ci()*). As expected, smaller ensemble sizes result in larger uncertainty (larger CIs) without exception. The results suggest that the uncertainty in especially ξ increases particularly strongly when ensemble size drops below 20, this effect is also seen for other latitude bands and in NDJFM (extended winter). Overall, the consistency of all three GEV parameters down to an ensemble size of 20 is very high.
The ensemble size is not the critical metric, however, since the GEV-fit robustness depends on the sample size, which depends on ensemble size but is not identical to it. Our setup, using 5yRx1d records for non-stationary GEV distribution fitting, has the following consequences for the sample size: The 1850-2100 record yields 50 5yRx1d values, resulting in 1000 values for each grid cell for a 20-member ensemble. The non-stationary GEV distribution requires estimation of five parameters (μ_0, μ_1, σ_0, σ_1, ξ). This means that we have, on average, a ratio of 200 sample values per estimated parameter for 5yRx1d in a 20 member ensemble. As a very first-order estimate, we might thus say that record breaking/shattering estimates based on fitted GEV distributions can be made with moderate ensemble sizes, as long as the requirement of a minimum of roughly 200 sample values per GEV parameter to be estimated is satisfied. The shape parameter ξ is the critical parameter for GEV goodness of fit, having the highest sensitivity to a decrease in sample size. This is in line with the known difficulty concerned with ξ-estimation [1].

S6 Supplementary section 6: Consistency GEV-based analytical solution in CMIP6

Fig. S18 adds to the CMIP6 results shown in main Fig. 2 by assessing the similarity of the estimated GEV parameters across models. We show the same metrics as in Fig. S17.
We see substantial variation between the models, but also many similarities. Firstly, the models agree well on μ (top panel) in the midlatitudes (black outline), and show a similar evolution between the early and the late 50-year window. It is of no surprise that the models differ in the absolute magnitude of the 5yRx1d μ values, and this variation is well constrained for midlatitudes. For the tropical latitude band (light grey outline), however, the two MPI models show remarkably low values, whereas the other three models lie closer to one another in a range that seems more feasible for the tropics. This most likely has to do with the known underestimation of tropical precipitation in MPI, and possibly a different location of the intertropical convergence zone [3]. The 95% CIs for μ are relatively consistent, however, we see a higher uncertainty for MPI-ESM1-2-HR and UKESM1-0-LL in the midlatitudes, most likely due to the

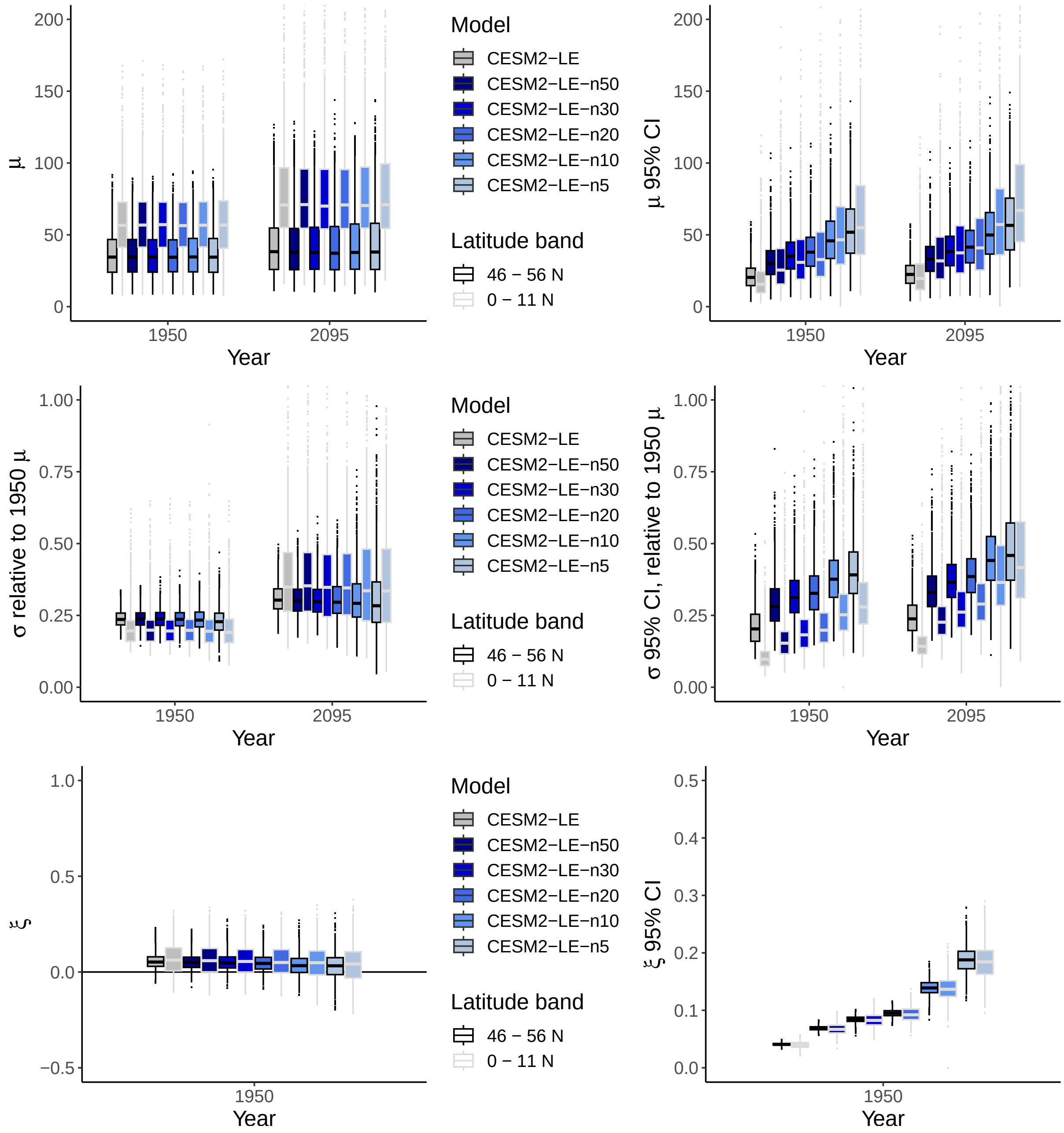

SI Figure S17: Sensitivity of fitted GEV parameters to CESM2 ensemble size, all for MJJAS. The full ensemble of 100 members is compared to randomly selected sub-ensembles of 50, 40, 30, 20, 10 and 5 members. Location parameter μ (top), scale parameter σ, normalised w.r.t. the 1950 μ-value (middle) and shape parameter ξ (bottom) are shown. Left panel shows 5yRx1d GEV parameter estimates for a latitude band in the midlatitudes (black outline) and one in the tropics (light grey outline) in 1950-1954 and 2095-2099 windows, right panel shows the magnitude of the 95% confidence intervals (CIs) around the mean for the respective parameters.

combination of the variable character of summer precipitation (convective) and the small ensemble size for these models (10 and 13 members).
σ (middle panel) is expressed as a fraction of the local 1950 μ value for ease of comparison, hence the smaller relative σ in the tropics than in the midlatitudes are due to the tropical mean precipitation being very high. CESM2-LE and UKESM1-0-LL show the highest relative σs overall, and the two MPI models the lowest, which, in combination with μ seems to suggest a systematically low and narrow extreme precipitation distribution in MPI models, and the opposite for CESM2-LE and UKESM1-0-LL. ACCESS-ESM1-5 lies in between. Finally, it stands out that CESM2-LE and UKESM-0-LL feature a

strong increase in σ between 1950 and 2095, primarily in the tropics. We explore this further in Fig. S19.
ξ estimates (bottom panel) are consistently small, apart from the large spread for UKESM1-0-LL tropics, which is most likely due to its small ensemble. For the midlatitudes, the majority of the models has small positive shape parameters, in line with the expectation for extreme precipitation distributions (fat right tail, no upper bound) [4]. The ξ estimates are less consistent across and within models for the tropics, indicating once again that ξ is a main driver of uncertainties in GEV distribution fits, and thereby in GEV-derived record-shattering probabilities. This is also in line with the apparent lower goodness of fit of the tails of GEV distributions for 5yRx1d in the tropics in general, which is likely related to the very long tail of the extreme precipitation distribution in the tropics, as further expanded on in Supplementary Sect. S7. In the 95% CI we see that the small ensembles of MPI-ESM1-2HR and UKESM1-0-LL result in substantially higher uncertainties in the ξ estimate for those models. As shown in Supplementary Sect. S5, ensembles of fewer than 20 members in our setup considerably decrease GEV goodness of fit due to high uncertainty in ξ, and MPI-ESM1-2HR and UKESM1-0-LL have ensembles smaller than 20 indeed (10 and 13).
As a final comparison, Fig. S19 shows the magnitude of μ and σ in 2100 relative to their 1950 values as a measure of distribution change. The relative changes are qualitatively remarkably consistent across models, with moderate but robust increases in both μ and σ. Models generally agree on larger relative changes in the tropical latitudes, but CESM2-LE and UKESM1-0-LL show particularly strong relative increases in the tropics. This suggests that the steep increase in record-shattering probability as well as the large role for variability increases that we report in the main text are partly dependent on the model specific extreme precipitation response. It is important to emphasise that not all models show the strong changes leading to instantaneous record-shattering probability increases. The differences in the extreme precipitation responses of the models are aligned with their differences in climate sensitivity [5]. Nonetheless, if such strong increases in extreme precipitation variability, corresponding to climate sensitivities on the high end of the spectrum, do become apparent in our real climate, strong instantaneous increases in record-shattering probability will as well.

S7 Supplementary section 7: Validation spatially correlated data sample generation

Fig. S20 provides a validation check of the method used to arrive at the semi-analytical solution for the regionally pooled record-shattering probability estimates, based on generated samples of spatially correlated GEV-distributed data (see main Sect. 4.6 for the method and main Sect. 2.5 and 2.6 for the results). It shows the spatial correlation matrices of the original MJJAS 5yRx1d data, generated MJJAS 5yRx1d data, and their difference for the three regions we focus on in the main text. The similarities of the patterns in the CESM2 and generated data matrices corroborate that the spatial correlation structure of the original data and the generated data are in very high agreement, as confirmed by the small magnitude of the differences. In Pakistan and Lagos the difference between the original and generated data shows no systematic bias; the mean of the differences is close to zero. In BNLG, the differences seem to be slightly skewed towards negative values, which suggests the spatial dependence in the generated data is a bit too low. This could lead to a slight overestimation of record-shattering probabilities. The bias is only very small, however, and its effect is not systematically visible in the results.

S8 Supplementary section 8: NDJFM behaviour in BNLG

As mentioned in the main text, we see a higher normalised μ-trend in local winter in the BNLG region, related to winter precipitation being mesoscale system-driven, having a large scale character and lower baseline variability. This in turn leads to an increase in record-shattering probability with climate change that is dominated by the mean trend, as Supplementary Fig. S21a demonstrates.

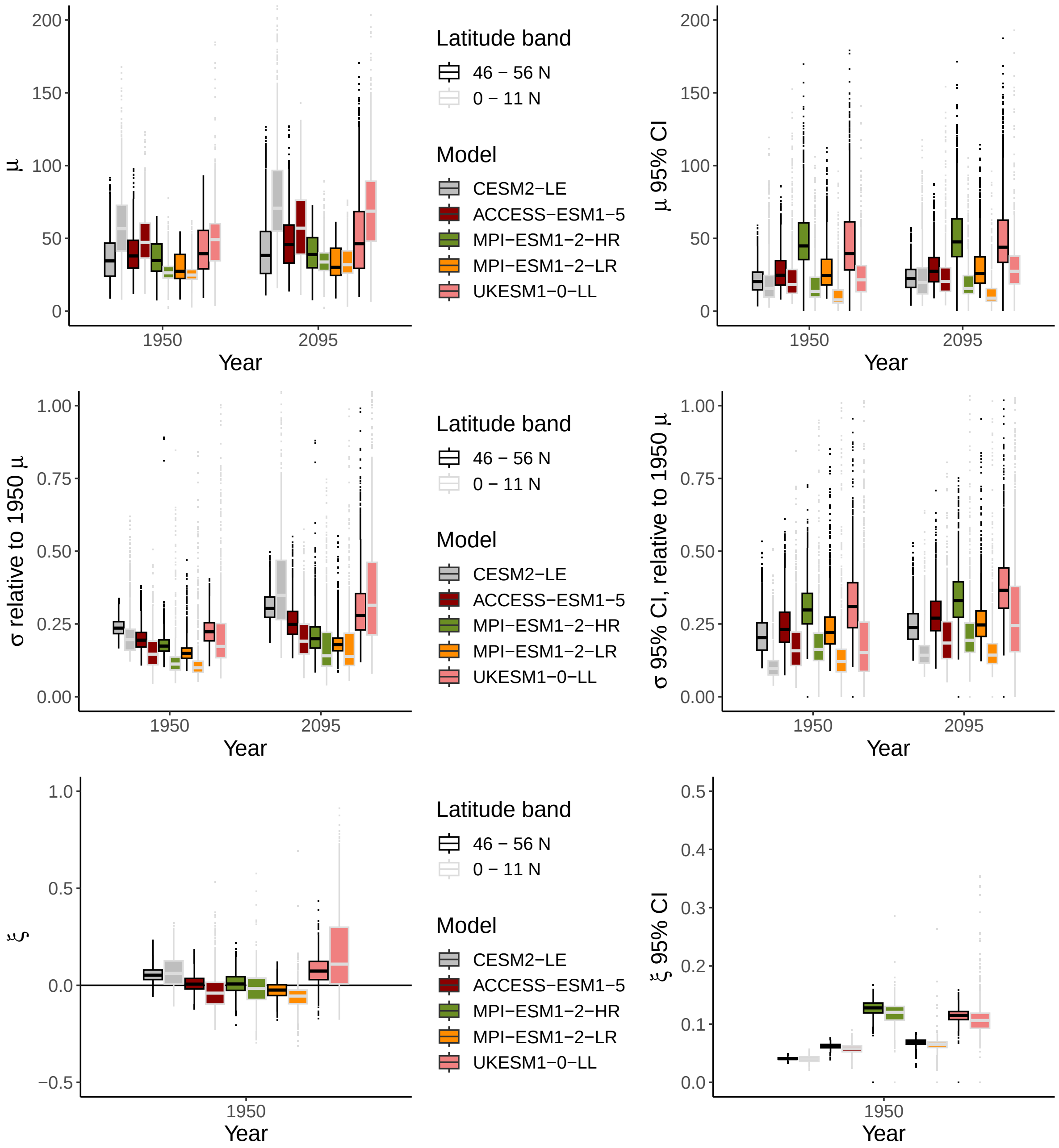

SI Figure S18: As Fig. S17 but here the GEV parameters of CESM2-LE are compared to those of four CMIP6 models.

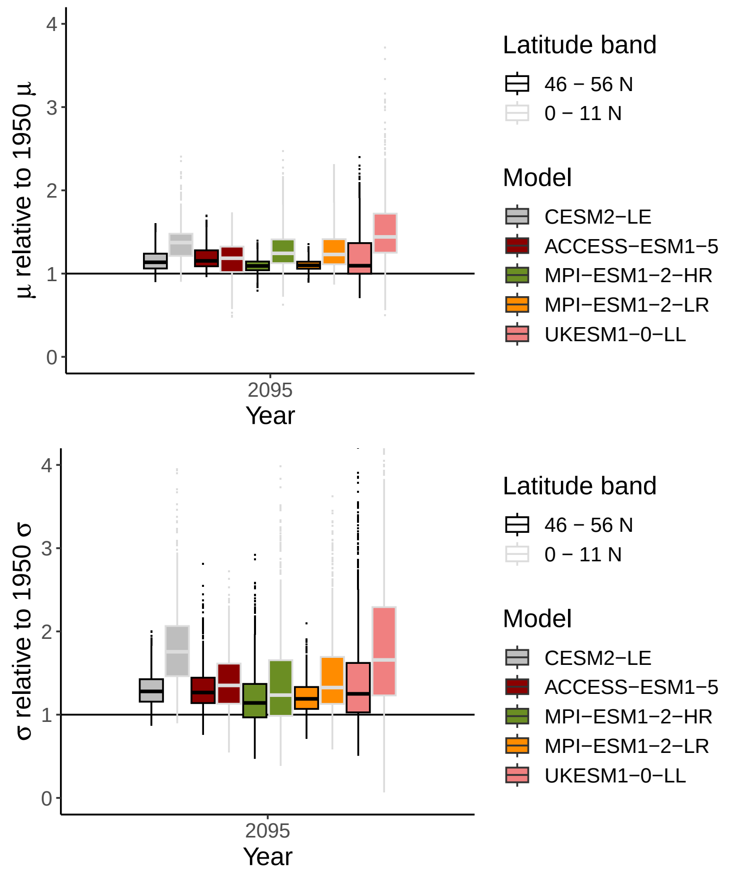

SI Figure S19: Change in 5yRx1d GEV parameter estimates in the 2095-2099 window relative to their 1950-1954 values for a latitude band in the midlatitudes (black outline) and one in the tropics (light grey outline) for CESM2-LE and four CMIP6 models.

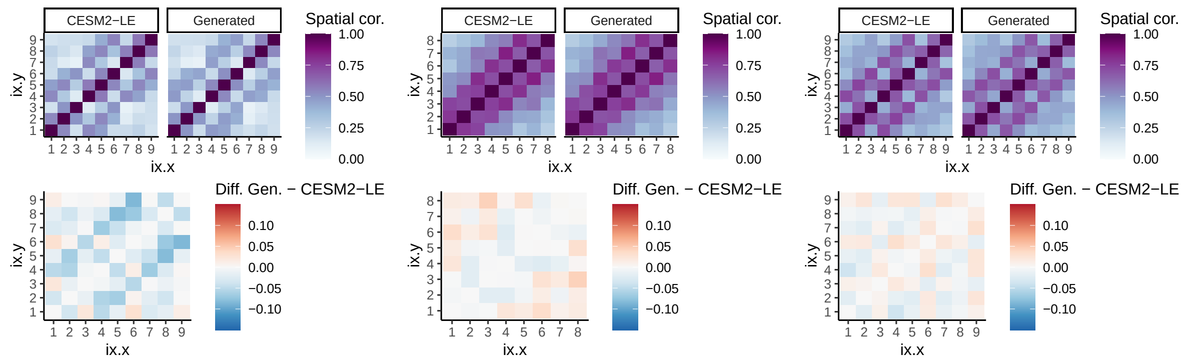

(a) BNLG (b) Pakistan (c) Lagos

SI Figure S20: Within-region spatial correlations of CESM2-LE data and semi-analytical generated data (top) and their difference (bottom) for MJJAS in the regions shown in main Fig. 3

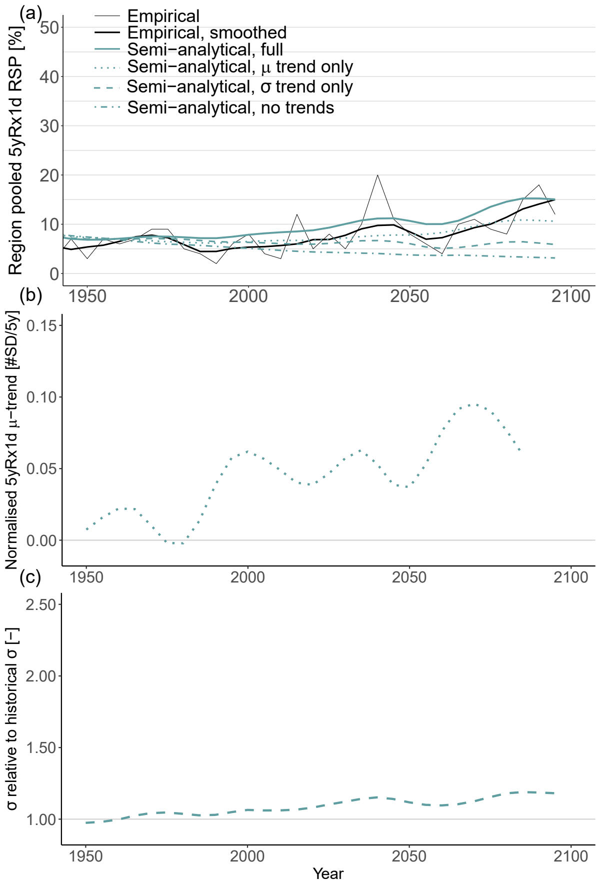

SI Figure S21: As main Fig. 3 but for BNLG NDJFM (local winter) only. a shows regionally pooled record-shattering probability including breakdown into μ and σ components. b and c show the normalised μ-trend and relative magnitude of σ.

Supplementary References

[1] Coles, S.: Extremes of Non-stationary Sequences, pp. 105–123. Springer, London (2001). https://doi.org/10.1007/978-1-4471-3675-0_6 . p. 106

[2] Zeder, J., Sippel, S., Pasche, O.C., Engelke, S., Fischer, E.M.: The effect of a short observational record on the statistics of temperature extremes. Geophysical Research Letters **50**(16), 2023–104090 (2023) https://doi.org/10.1029/2023GL104090

[3] Abdelmoaty, H.M., Papalexiou, S.M., Rajulapati, C.R., AghaKouchak, A.: Biases beyond the mean in cmip6 extreme precipitation: A global investigation. Earth's Future **9**(10), 2021–002196 (2021) https://doi.org/10.1029/2021EF002196

[4] Ragulina, G., Reitan, T.: Generalized extreme value shape parameter and its nature for extreme precipitation using long time series and the bayesian approach. Hydrological Sciences Journal **62**(6), 863–879 (2017) https://doi.org/10.1080/02626667.2016.1260134

[5] Meehl, G.A., Senior, C.A., Eyring, V., Flato, G., Lamarque, J.-F., Stouffer, R.J., Taylor, K.E., Schlund, M.: Context for interpreting equilibrium climate sensitivity and transient climate response from the cmip6 earth system models. Science Advances **6**(26), 1981 (2020) https://doi.org/10.1126/sciadv.aba1981
